# Prenatal Phthalate, Perfluoroalkyl Acid, and Organochlorine Exposures and Term Birth Weight in Three Birth Cohorts: Multi-Pollutant Models Based on Elastic Net Regression

**DOI:** 10.1289/ehp.1408933

**Published:** 2015-06-26

**Authors:** Virissa Lenters, Lützen Portengen, Anna Rignell-Hydbom, Bo A.G. Jönsson, Christian H. Lindh, Aldert H. Piersma, Gunnar Toft, Jens Peter Bonde, Dick Heederik, Lars Rylander, Roel Vermeulen

**Affiliations:** 1 Division of Environmental Epidemiology, Institute for Risk Assessment Sciences, Utrecht University, Utrecht, the Netherlands; 2 Division of Occupational and Environmental Medicine, Lund University, Lund, Sweden; 3 Laboratory for Health Protection Research, National Institute for Public Health and the Environment (RIVM), Bilthoven, the Netherlands; 4 Danish Ramazzini Center, Department of Occupational Medicine, Aarhus University Hospital, Aarhus, Denmark; 5 Department of Occupational and Environmental Medicine, Copenhagen University Hospital, Bispebjerg, Copenhagen, Denmark; 6 Julius Center for Health Sciences and Primary Care, University Medical Center Utrecht, Utrecht, the Netherlands

## Abstract

**Background:**

Some legacy and emerging environmental contaminants are suspected risk factors for intrauterine growth restriction. However, the evidence is equivocal, in part due to difficulties in disentangling the effects of mixtures.

**Objectives:**

We assessed associations between multiple correlated biomarkers of environmental exposure and birth weight.

**Methods:**

We evaluated a cohort of 1,250 term (≥ 37 weeks gestation) singleton infants, born to 513 mothers from Greenland, 180 from Poland, and 557 from Ukraine, who were recruited during antenatal care visits in 2002‒2004. Secondary metabolites of diethylhexyl and diisononyl phthalates (DEHP, DiNP), eight perfluoroalkyl acids, and organochlorines (PCB-153 and *p,p´-*DDE) were quantifiable in 72‒100% of maternal serum samples. We assessed associations between exposures and term birth weight, adjusting for co-exposures and covariates, including prepregnancy body mass index. To identify independent associations, we applied the elastic net penalty to linear regression models.

**Results:**

Two phthalate metabolites (MEHHP, MOiNP), perfluorooctanoic acid (PFOA), and *p,p´-*DDE were most consistently predictive of term birth weight based on elastic net penalty regression. In an adjusted, unpenalized regression model of the four exposures, 2-SD increases in natural log–transformed MEHHP, PFOA, and *p,p´-*DDE were associated with lower birth weight: –87 g (95% CI: –137, –340 per 1.70 ng/mL), –43 g (95% CI: –108, 23 per 1.18 ng/mL), and –135 g (95% CI: –192, –78 per 1.82 ng/g lipid), respectively; and MOiNP was associated with higher birth weight (46 g; 95% CI: –5, 97 per 2.22 ng/mL).

**Conclusions:**

This study suggests that several of the environmental contaminants, belonging to three chemical classes, may be independently associated with impaired fetal growth. These results warrant follow-up in other cohorts.

**Citation:**

Lenters V, Portengen L, Rignell-Hydbom A, Jönsson BA, Lindh CH, Piersma AH, Toft G, Bonde JP, Heederik D, Rylander L, Vermeulen R. 2016. Prenatal phthalate, perfluoroalkyl acid, and organochlorine exposures and term birth weight in three birth cohorts: multi-pollutant models based on elastic net regression. Environ Health Perspect 124:365–372; http://dx.doi.org/10.1289/ehp.1408933

## Introduction

Reduced birth weight is associated with increased short- and long-term morbidities and mortality (McIntire et al. 1999; Risnes et al. 2011). This is in line with the developmental origins of health and disease hypothesis: that *in utero* and early-life stressors can impact certain chronic disease risks throughout the life course (Gluckman and Hanson 2004). There is a growing although inconsistent body of evidence that some environmental exposures, including background levels of human-made chemicals, are risk factors for impaired fetal growth (Nieuwenhuijsen et al. 2013; Windham and Fenster 2008).

Numerous studies have investigated the relationship between organochlorine compounds, including polychlorinated biphenyls (PCBs) and dichlorodiphenyltrichloroethane (DDT), or its main metabolite dichlorodiphenyldichloroethylene (*p*,*p*´-DDE), and markers of fetal growth. A recent pooled analysis of 14 European study populations showed that prenatal exposure to PCB-153 was associated with a significant decrease in birth weight, and *p*,*p*´-DDE with a nonsignificant decrease (Casas et al. 2015). A smaller though substantial number of studies have investigated perfluoroalkyl and polyfluoroalkyl substances [PFASs; also known as perfluorinated compounds (PFCs)] in relation to birth weight. Perfluorooctanoic acid (PFOA) and perfluorooctane sulfonate (PFOS) have been studied most extensively (e.g., Darrow et al. 2013; Fei et al. 2007; Washino et al. 2009); perfluorohexane sulfonate (PFHxS), perfluorononanoic acid (PFNA), and perfluoroundecanoic acid (PFUA) have also been assessed (Chen et al. 2012; Maisonet et al. 2012). Associations have generally been null or in the direction of a negative effect on size metrics at birth, although inconsistent across PFASs. The few studies on phenols and phthalates have yielded inconsistent results (e.g., Philippat et al. 2012; Wolff et al. 2008).

Measurements in maternal biospecimens, cord blood, and amniotic fluid indicate widespread fetal exposure to these environmental contaminants (Braun et al. 2012; Casas et al. 2015; Glynn et al. 2012; Jensen et al. 2012). Diet is the primary source of adult exposure, via lipid-rich food or food packaging. Although PCBs and DDT have been banned or restricted, exposure will continue due to their environmental persistence. PFASs are surfactants with diverse industrial and commercial applications. Some are being phased out (e.g., PFOS, PFOA), but biomonitoring suggests they are being replaced by other PFASs (Glynn et al. 2012). Diethylhexyl phthalate (DEHP) and diisononyl phthalate (DiNP) are high-molecular-weight phthalates used as plasticizers in various products including polyvinyl chloride, which, despite their relatively rapid elimination, have high detection rates.

Previous studies have rarely evaluated more than one chemical class simultaneously. In the present study of mother–infant pairs from Greenland, Poland, and Ukraine, metabolites of two phthalates (DEHP, DiNP), eight PFASs, PCB-153, and *p*,*p*´-DDE were measured in serum of pregnant women. Our objective was to characterize associations between the multiple, correlated biomarkers of prenatal exposure and birth weight. We used a variable selection method, elastic net regression, in tandem with unpenalized regression models, to estimate the independent effects of exposures.

## Methods

*Study populations.* Pregnant women were enrolled between June 2002 and May 2004 during routine antenatal care visits at *a*) local hospitals or clinics in 19 municipalities and settlements throughout Greenland, *b*) three hospitals and eight antenatal clinics in Kharkiv, Ukraine, and *c*) a large central hospital in Warsaw, Poland. Eligible participants were at least 18 years old and born in the country of study. At enrollment, women participated in an interview on time to pregnancy and were invited to donate a nonfasting venous blood sample. The study was approved by the local ethics committees, and written informed consent was obtained from all participating women. Details on the INUENDO cohort study design and data collection have been published (Jönsson et al. 2005; Toft et al. 2005; Wojtyniak et al. 2010). Briefly, 1,710 couples were interviewed, with a participation rate of 90% in Greenland, 68% in Poland, and 26% in Ukraine; of those, a blood sample was collected and available for analysis for 96%, 55%, and 96% of women, respectively. Blood samples were collected on average later during pregnancy for participants in Poland than in Greenland and Ukraine (median of 33, 25, and 23 gestational weeks, respectively).

There were 1,321 mother–singleton infant pairs with complete exposure and birth weight data. Twenty-three pairs (1.7%) with missing data on maternal age, body mass index (BMI), parity, gestational duration, or infant sex were further excluded.

*Outcome and covariate ascertainment.* Birth outcome data were extracted from hospital maternity records by medical personnel.

Gestational age was assigned based on self-reported date of last menstrual period. The outcome of interest, birth weight, was analyzed for only infants delivered at term (≥ 37 completed weeks of gestation, *n* = 1,250) (Wilcox 2001). We did not analyze preterm birth because of the limited power (*n* = 48).

Information on sociodemographic and lifestyle factors and reproductive history was ascertained during the baseline interview. Serum cotinine levels were used as an indicator of exposure to tobacco smoke during pregnancy. Prepregnancy BMI was calculated using self-reported weight and height.

*Exposure assessment.* PFASs and phthalates, along with cotinine and vitamin D, were simultaneously determined in 100-μL aliquots of serum by liquid chromatography–tandem mass spectrometry, following an optimized protocol based on Lindh et al. (2012) and Specht et al. (2014). Secondary oxidative metabolites of DEHP [mono(2-ethyl-5-hydroxyhexyl) phthalate (MEHHP, alternatively 5OH-MEHP), mono(2-ethyl-5-oxohexyl) phthalate (MEOHP, alternatively 5oxo-MEHP), mono(2-ethyl-5-carboxypentyl) phthalate (MECPP, alternatively 5cx-MEPP)] and DiNP [mono(4-methyl-7-hydroxyloctyl) phthalate (MHiNP, alternatively 7OH-MMeOP), mono(4-methyl-7-oxo-octyl) phthalate (MOiNP, alternatively 7oxo-MMeOP), and mono(4-methyl-7-carboxyheptyl) phthalate (MCiOP, alternatively 7cx-MMeHP)] were analyzed. Analyzed perfluoroalkyl acids (PFAAs), a subset of PFASs, included perfluoroheptanoic acid (PFHpA), PFHxS, PFOS, PFOA, PFNA, perfluorodecanoic acid (PFDA), perfluoroundecanoic acid (PFUnDA), and perfluorododecanoic acid (PFDoDA).

PCB-153 and *p,p´*-DDE were analyzed by gas chromatography–mass spectrometry, as previously described (Jönsson et al. 2005). Lipids were determined by enzymatic methods, and lipid adjustment of organochlorines was based on total lipids, calculated as 1.13 + 1.31 × (triglycerides + cholesterol) (Rylander et al. 2006). Limits of detection (LODs) ranged from 0.005 to 0.2 ng/mL, and the interassay coefficients of variation in quality control samples were between 6% and 21% (median, 13.5%) (see Supplemental Material, Table S1).

*Statistical analysis.* In the case of multicollinearity, multiple linear regression models may yield unreliable parameter estimates. Therefore, to assess which exposures are associated with the outcome while simultaneously adjusting for other exposures, we used elastic net regression modeling (Equation 1): generalized linear models fit with a hybrid of the lasso and ridge penalty functions (Friedman et al. 2010; Zou and Hastie 2005). Ridge penalizes the square of the regression coefficients for the predictors, shrinking coefficients from correlated predictors proportionally toward zero. Lasso imposes a penalty on the absolute value of the coefficients, shrinking coefficients by a constant factor, and can select a subset of predictors by shrinking coefficients for the least predictive predictors exactly to zero. Whereas ridge retains all predictors, and lasso tends to select only one predictor from a group of correlated predictors, elastic net can perform selection while enabling the inclusion of collinear predictors in the final model.

A set of elastic net coefficients (**β**_EN_) are estimated by minimizing the residual sum of squares, subject to a weighted sum of the lasso and ridge penalties:


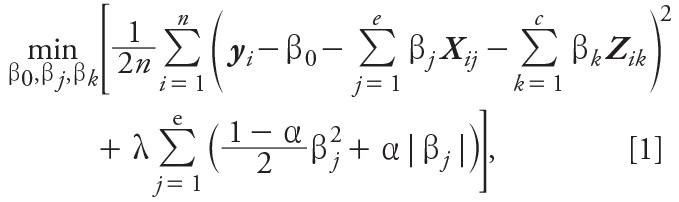


where 0 ≤ α ≤ 1 and λ ≥ 0; and ***y****_i_* is the *n ×* 1 vector of outcome values for *i* = 1,*…*,*n* participants, β_0_ is the intercept, β_j_ and β_k_ are vectors of regression coefficients that correspond to the main effects of respectively ***X****_ij_*, the *n* × *e* matrix of *j* = 1,…,*e* standardized exposures, and ***Z****_ik_*, the *n × c* matrix of *k* = 1,…,*c* standardized potentially confounding covariates. To fully adjust for potential confounders, we selected confounders *a priori*, without subjecting them to variable selection, and included them in the elastic net regression models as unpenalized variables. Thus, α controls the balance between the lasso (α = 1) and ridge (α = 0) penalties, and λ represents the penalty (or regularization) parameter, with the degree of shrinkage increasing as λ increases for a given α value.

We used cross-validation (CV) to determine the optimal degree of penalization. We tested models over a grid of α and λ sequences, and selected the combination which yielded the minimum mean-squared error (MSE) of prediction from repeated 10-fold CV. CV was repeated 100 times, each time with different data partitions, to achieve more stable selection than a single CV. CV has been shown in simulations to have good sensitivity (true positive rate) of selection, yet can suffer from inflated false discovery rates (Sabourin et al. 2015). Therefore, to complement CV and to aid in statistical inference of the results, we also estimated the more stringent, at a loss of power, covariance test *p*-values of Lockhart et al. (2014); this assesses the significance of each exposure upon its inclusion in the model as the degree of penalization decreases, conditional on other important predictors having been included in the model first.

For the subset of exposures selected via elastic net, and inferred to be important—giving consideration to selection consistency across adjustment models, the covariance test, and the magnitude of the coefficients—we refit multiple-exposure ordinary least squares (OLS) regression models to obtain unpenalized, mutually adjusted coefficient estimates.

Before modeling, exposure variables were natural log (ln)–transformed to reduce the influence of outliers. Data below the LOD (0‒27%) were singly imputed from a log-normal probability distribution, dependent on the study population and observed values for other contaminants, and allowing residual variances to vary by population (Lubin et al. 2004). For regression models, we mean-centered all predictor variables and scaled continuous variables by two times their respective standard deviations (SD) (Gelman 2008) to impart variables with the same prior importance in penalized regressions, and to improve the comparability of coefficients for continuous and binary predictors.

We identified the minimal sufficient adjustment set using a directed acyclic graph (see Supplemental Material, Figure S1): study population (an indicator variable for participant location), maternal age (27‒31, 32‒45, vs. 18‒26 years; categories based on the lowest Akaike Information Criterion of birth weight on age), prepregnancy BMI (kilograms per meter squared), and parity (≥ 1 vs. 0). Gestational age at birth (weeks) was added in a separate model because it may represent an intermediate variable (or collider)—though it presumably has no direct effect on measured maternal serum levels, it affects the unmeasured cumulative exposure of the fetus, and may also be causally affected by exposures—and there is no consensus on how to adjust for this variable in the literature. In further adjusted models, additional potential confounders were added, possibly leading to over- or unnecessary adjustment: infant sex (female vs. male), maternal height (centimeters), alcohol consumption around the period of conception (≥ 7 vs. < 7 drinks/week), maternal serum cotinine (nanograms per milliliter), and maternal serum vitamin D (nanograms per milliliter). Because of the large proportion of missing data for maternal education, seafood consumption, and season of blood sampling, these variables were included only in a sensitivity analysis.

For comparison, we assessed associations between single exposures and term birth weight with OLS models, controlling the false discovery rate (FDR) at 5% (Benjamini and Hochberg 1995). In sensitivity analyses, we assessed the linearity of adjusted single-exposure–outcome relationships using generalized additive models (GAMs), smoothing the exposure term with a penalized regression spline. We also tested for effect measure modification by study population, infant sex, prepregnancy BMI (< 25, ≥ 25 kg/m^2^), and smoking [< 5, ≥ 5 ng/mL cotinine (Benowitz et al. 2009)] in stratified analyses and by introducing product interaction terms between potential modifiers and selected exposures in OLS models. Recognizing that using lipid-standardized organochlorine levels may lead to overadjustment if lipids represent an intermediate, coefficients for wet weight PCB-153 and *p,p´*-DDE were additionally estimated, with total lipids included as a covariate (Schisterman et al. 2005). Lipids were not included in the primary multiple-exposure models to avoid overadjustment of the phthalate and PFAS coefficients. Additional models were tested including the molar sum of both DEHP and DiNP metabolites instead of their individual metabolites. The default statistical significance level was set at α = 0.05. We analyzed data using R version 3.0.3; (R Core Team 2014), and fit elastic net models using the *glmnet* package (Friedman et al. 2010).

## Results

Participant characteristics varied somewhat across study populations (Table 1). Women from Poland were older than women from Greenland and Ukraine, and more women from Greenland were multiparous, overweight, or obese, and had higher cotinine levels, indicating exposure to tobacco smoke during pregnancy. The mean birth weight was 3,651, 3,530, and 3,302 g for term newborns from Greenland, Poland, and Ukraine, respectively, and 19 infants had a low birth weight (< 2,500 g).

**Table 1:** Characteristics of the study populations (2002–2004, *n* = 1,250) [*n* (%) or mean ± SD].

Characteristic	Greenland (*n* = 513)	Warsaw, Poland (*n* = 180)	Kharkiv, Ukraine (*n* = 557)
Maternal age at delivery (years)
18–24	235 (45.8)	12 (6.7)	299 (53.7)
25–29	119 (23.2)	110 (61.1)	164 (29.4)
30–34	82 (16.0)	55 (27.8)	76 (13.6)
35–45	77 (15.0)	8 (4.4)	18 (3.2)
Prepregnancy BMI (kg/m^2^)
< 18.5	14 (2.7)	11 (6.1)	79 (14.2)
18.5–24.9	306 (59.6)	154 (85.6)	410 (73.6)
25.0–29.9	136 (26.5)	13 (7.2)	56 (10.1)
≥ 30	57 (11.1)	2 (1.1)	12 (2.2)
Maternal height (cm)	162.0 ± 6.8	166.3 ± 5.2	165.28 ± 6.0
Parity
0	161 (31.4)	153 (85.0)	440 (79.0)
1	153 (29.8)	24 (13.3)	95 (17.1)
2	104 (20.3)	2 (1.1)	12 (2.2)
≥ 3	95 (18.5)	1 (0.6)	10 (1.8)
Serum cotinine (ng/mL)
< 5.0	208 (40.5)	178 (98.9)	465 (83.5)
5.0–49.9	87 (17.0)	2 (1.1)	54 (9.7)
≥ 50.0	218 (42.5)	0 (0.0)	38 (6.8)
Maternal education
No postsecondary	244 (50.9)	8 (4.4)	221 (39.7)
Some postsecondary	235 (49.1)	172 (95.6)	335 (60.3)
Missing	34	0	1
Alcohol intake (drinks/week)^*a*^
< 7	450 (87.7)	169 (93.9)	553 (99.3)
≥ 7	63 (12.3)	11 (6.1)	4 (0.7)
Fish or seafood (days/week)	1.87 ± 1.53	1.28 ± 1.06	1.08 ± 1.53
Missing	12	10	60
Serum vitamin D (ng/mL)	17.56 ± 9.51	28.34 ± 11.73	22.46 ± 10.73
Timing of blood sampling (gestational weeks)
1–13	66 (13.3)	1 (0.6)	157 (28.9)
14–26	229 (46.1)	11 (6.2)	156 (28.7)
≥ 27	202 (40.6)	166 (93.3)	231 (42.5)
Missing	16	2	13
Season of blood sampling
October–March	274 (53.5)	135 (75.8)	353 (64.5)
April–September	238 (46.5)	43 (24.2)	194 (35.5)
Missing	1	2	10
Infant sex
Female	237 (46.2)	92 (51.1)	264 (47.4)
Male	276 (53.8)	88 (48.9)	293 (52.6)
Term birth weight (g)
< 2,500	11 (2.1)	1 (0.6)	7 (1.3)
2,500–2,999	44 (8.6)	18 (10.0)	98 (17.6)
3,000–3,499	142 (27.7)	71 (39.4)	269 (48.3)
3,500–3,999	175 (34.1)	66 (36.7)	158 (28.4)
≥ 4,000	141 (27.5)	24 (13.3)	25 (4.5)
Gestational age (weeks)	39.85 ± 1.34	39.53 ± 1.25	39.21 ± 1.00
^***a***^During the period around conception.			

**Figure 1:**
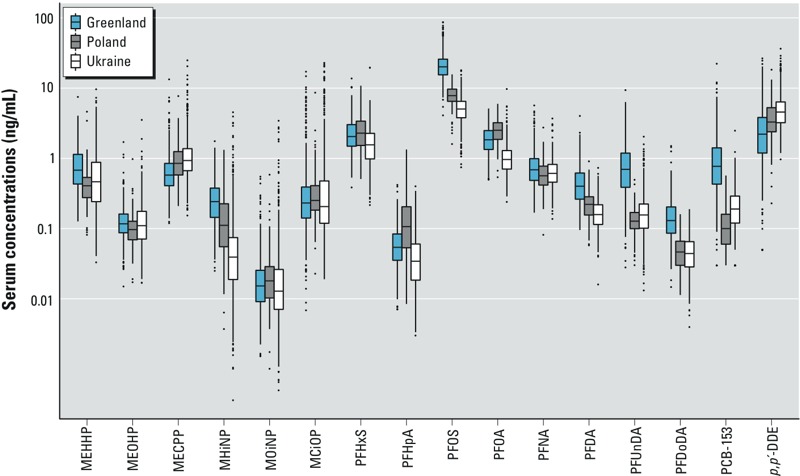
Box plots of distributions of exposure biomarker concentrations per study population. Horizontal lines correspond to medians, and boxes to the 25th–75th percentiles; whiskers extend to data within the interquartile range times 1.5, and data beyond this are plotted as dots. Wet weight concentrations are presented for PCB-153 and *p,p’*-DDE.

**Figure 2:**
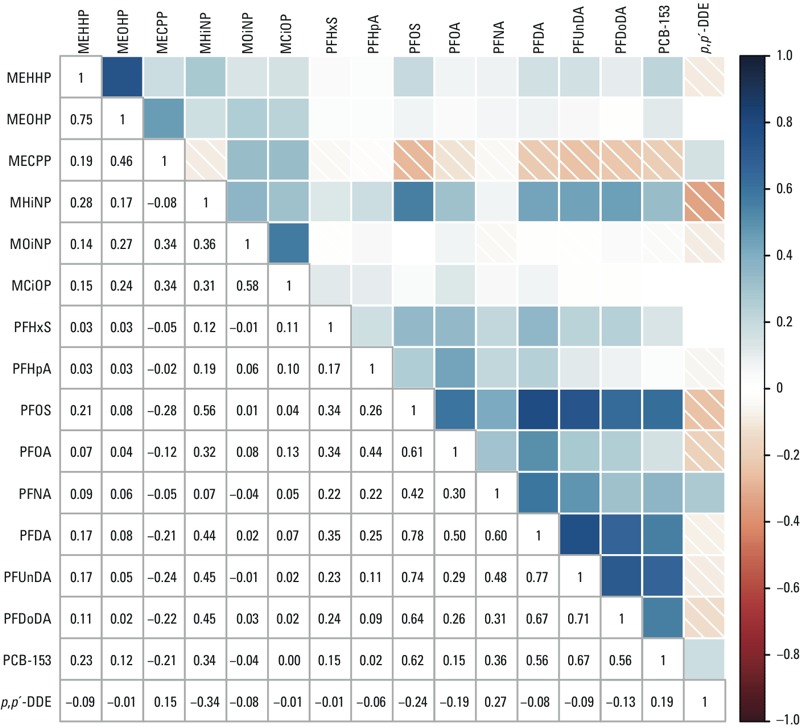
Spearman correlation coefficients between exposure biomarkers. The color intensity of shaded boxes indicates the magnitude of the correlation. Blue indicates a positive correlation, and red with white diagonal lines indicates a negative correlation.

All 16 exposure biomarkers were quantifiable in at least 72% of serum samples, and 11 were quantifiable in at least 98% (see Supplemental Material, Table S1). Exposure distributions differed across study populations for nearly all contaminants (Figure 1; see also Supplemental Material, Table S1). For 9 contaminants, median concentrations were highest for women from Greenland. Pooled across countries, wet weight concentrations were highest for PFOS, followed by *p,p´*-DDE (median, 8.43 and 3.39 ng/mL). Spearman correlation coefficients between exposures ranged from –0.34 to 0.78 (Figure 2), and were generally similar across the three study populations (see Supplemental Material, Table S2).

We observed several associations between birth weight, selected exposures, and characteristics of the total study population consistent with the literature (see Supplemental Material, Table S3). For instance, maternal age exhibited an inverted U-shaped association and parity a positive association with term birth weight, whereas maternal age was positively associated and parity negatively associated (for some, nonsignificantly) with persistent contaminant concentrations.

In the primary variable selection analysis, with minimal sufficient adjustment, eight exposures were selected (β ≠ 0; seven exposures with β_EN_ > |1.0|) in the multiple-exposure elastic net regression of term birth weight (Table 2). MEHHP, MOiNP, PFOA, and *p,p´-*DDE were consistently selected in elastic net modeling, and exhibited the largest magnitude beta coefficients, across the three adjusted models. In the covariance test, *p,p´-*DDE was consistently significant, and MEHHP was significant in two models (Table 2). Upon inputting the four exposures into multiple-exposure unpenalized models, β_OLS_ estimates for MEHHP and *p,p´-*DDE were negative and statistically significant across models, whereas estimates for PFOA were negative but significant only in the further adjusted model, and the estimates for MOiNP were positive but did not reach statistical significance (Table 3). These four exposures were not highly correlated (*r*_s_ = –0.19 to 0.14) (Figure 2). PFHxS, PFNA, and PFDoDA were also variably selected with nontrivial coefficients (β_EN_ > |1.0|) across adjusted models (Table 2). These exposures and the four primary-selected exposures showed some moderate correlations (*r*_s_ ≤ |0.34|; variance inflation factors 1.04‒2.17 for exposure terms), yielding potentially less reliable multiple-exposure β_OLS_ estimates for differences in term birth weight (grams) when added to the four-exposure model in turn: per 2-SD increase in ln-transformed PFHxS, –19.68 [95% confidence interval (CI): –74.20, 34.83]; PFNA, –17.34 (95% CI: –77.47, 42.78); PFDoDA, –56.23 (95% CI: –125.05, 12.59).

**Table 2:** Multiple-exposure elastic net penalized regression models*^a^* (β_EN_) for term birth weight.

Potential predictor (increment)	Adjusted	Plus gestational age	Further adjusted
ln-MEHHP (1.70 ng/mL)	–64.67^*b*^	–59.43^*b*^	–48.61
ln-MEOHP (1.29 ng/mL)	–0.15	0	0
ln-MECPP (1.42 ng/mL)	0	0	0
ln-MHiNP (2.74 ng/mL)	0	0	0
ln-MOiNP (2.22 ng/mL)	23.81	22.26	16.31
ln-MCiOP (2.32 ng/mL)	0	0	0
ln-PFHxS (1.24 ng/mL)	–3.49	0	0
ln-PFHpA (1.84 ng/mL)	0	0	0
ln-PFOS (1.60 ng/mL)	0	0	0
ln-PFOA (1.18 ng/mL)	–11.51	–10.11	–38.82
ln-PFNA (1.03 ng/mL)	–7.05	–7.69	0
ln-PFDA (1.40 ng/mL)	0	0	0
ln-PFUnDA (2.10 ng/mL)	0	0	0
ln-PFDoDA (1.67 ng/mL)	–22.56	0	0
ln-PCB-153 (2.43 ng/g)	0	0	0
ln-*p,p´*-DDE (1.82 ng/g)	–106.39^*b*^	–76.63^*b*^	–47.02^*b*^
Regression coefficients (β_EN_) represent the change in mean birth weight (g) for term infants per increment: a 2-SD increase in ln-transformed exposure biomarker levels. β_EN_ for the modeled, unpenalized covariates are not shown. ^***a***^The cross-validated optimum penalization was α = 1.00, λ = 3.32 (MSE = 205,061) for the adjusted model (minimal sufficient set: study population, maternal age, prepregnancy BMI, and parity); α = 1.00, λ = 3.32 (MSE = 177,179) for the model additionally adjusted for gestational age; and α = 0.98, λ = 2.46 (MSE = 166,329) for the further adjusted model (plus infant sex, maternal height, alcohol consumption, serum cotinine, and vitamin D). All models, *n *= 1,250. ^***b***^Covariance test (Lockhart et al. 2014) *p *< 0.05: *p*-values were 0.002, < 0.001, and 0.345 for MEHHP and < 0.001, 0.017, 0.046 for *p,p´*-DDE in the minimally adjusted, plus gestational age, and further adjusted models, respectively. *p* = 0.071 for PFOA in the further adjusted model; all other *p*-values were > 0.10.			

**Table 3:** Multiple-exposure unpenalized linear regression models for the exposures selected via elastic net regression and term birth weight [β_OLS_ (95% CI)].

Predictor (increment)	Adjusted	*p*‑Value	Plus gestational age	*p*‑Value	Further adjusted	*p*‑Value
ln-MEHHP (1.70 ng/mL)	–86.75 (–139.18, –34.32)	0.001	–83.94 (–132.68, –35.19)	0.001	–70.22 (–117.59, –22.85)	0.004
ln-MOiNP (2.22 ng/mL)	45.85 (–4.84, 96.54)	0.076	45.62 (–1.51, 92.74)	0.058	37.64 (–7.99, 83.27)	0.106
ln-PFOA (1.18 ng/mL)	–42.77 (–108.19, 22.65)	0.200	–41.02 (–101.83, 19.80)	0.186	–63.77 (–122.83, –4.71)	0.035
ln-*p,p´*-DDE^*a*^ (1.82 ng/g)	–134.73 (–191.93, –77.53)	< 0.001	–100.75 (–154.13, –47.36)	< 0.001	–66.70 (–119.38, –14.02)	0.013
Population
Poland	–40.24 (–133.58, 53.11)	0.398	4.84 (–82.16, 91.85)	0.913	–93.16 (–183.72, –2.60)	0.044
Ukraine	–218.89 (–300.66, –137.13)	< 0.001	–142.98 (–219.73, –66.22)	< 0.001	–256.90 (–338.77, –175.02)	< 0.001
Maternal age (years)
27–31	88.14 (23.05, 153.24)	0.008	80.01 (19.48, 140.53)	0.010	65.44 (6.74, 124.14)	0.029
32–45	30.53 (–42.52, 103.57)	0.413	38.08 (–29.83, 105.99)	0.272	37.19 (–28.83, 103.21)	0.270
BMI (8.62 kg/m^2^)	209.00 (155.53, 262.46)	< 0.001	181.37 (131.51, 231.22)	< 0.001	194.00 (145.42, 242.58)	< 0.001
Parity: multiparous	58.72 (–3.63, 121.06)	0.065	76.33 (18.32, 134.34)	0.010	85.92 (29.39, 142.44)	0.003
Gestational age (2.45 weeks)			343.78 (295.61, 391.94)	< 0.001	330.43 (283.70, 377.15)	< 0.001
Infant sex: female					–115.40 (–160.38, –70.43)	< 0.001
Maternal height (12.93 cm)					135.83 (88.38, 183.28)	< 0.001
Alcohol: ≥ 7 drinks/week					34.43 (–61.76, 130.62)	0.483
Cotinine (113.51 ng/mL)					–140.41 (–191.92, –88.89)	< 0.001
Vitamin D (22.05 ng/mL)					18.77 (–29.39, 66.93)	0.445
Regression coefficients (β_OLS_) represent the change in mean birth weight (g) for term infants per increment: a 2-SD increase in ln-transformed exposure biomarker or untransformed continuous covariate levels, or per category for categorical covariates. Reference categories are population, Greenland; maternal age, 18–26 years; parity, nulliparous; infant sex, male; alcohol, < 7 drinks/week (around the time of conception). Variance inflation factors for exposure terms ranged from 1.04 to 1.74. ^***a***^β_OLS_ for models including wet weight *p,p’*-DDE (ng/mL), adjusted for total lipids: –134.22 (95% CI: –191.43, –77.02), –99.91 (95% CI: –153.30, –46.52), –67.16 (95% CI: –119.80, –14.51).						

The magnitude of associations (β_EN_ and β_OLS_) generally decreased slightly upon adjustment for gestational age, and more so upon adjustment for further covariates, with the notable exception of PFOA, which exhibited a more pronounced reduction in further adjusted models (Table 3). The estimated associations for contaminants (–135 to around 40 g per 2-SD increase in levels) were of a similar magnitude as some other predictors of term birth weight, including parity, infant sex, and maternal smoking (specifically, a 2-SD increase in cotinine). When summed phthalate metabolites instead of individual metabolites were included, ΣDEHPom was selected (β_EN_ = –36.94; multiple-exposure β_OLS_ = –65.43; 95% CI: –116.06, –14.80 per 1.27 mol/mL) and ΣDiNPom was not selected.

In the comparative analysis with single-exposure OLS regression models, nine exposures were significantly associated (FDR *q* < 0.05) with term birth weight in the primary adjusted model, of which four were also selected (β_EN_ > |1.0|) in the elastic net model. In the models additionally adjusted for gestational age, four were selected in the OLS and five in the elastic net, and three in both models. In the further adjusted OLS and elastic net models, three of the same exposures were selected (MEHHP, PFOA, and *p,p´*-DDE), and an additional exposure (MOiNP) was selected in the elastic net model (see Supplemental Material, Table S4).

There was some evidence, although nonsignificant (interaction *p*-value > 0.05), of effect measure modification by study population for MOiNP and PFOA, because the direction of effect estimates was heterogeneous. There was no evidence that infant sex modified associations. Greater reductions in birth weight were observed for overweight/obese compared with normal-prepregnancy-weight women for *p,p´*-DDE (interaction *p*-value = 0.03) and for smokers compared with nonsmokers for MEHHP and PFOA (interaction *p*-values = 0.03) (see Supplemental Material, Table S5).

There was significant nonlinearity for the overall exposure–outcome relationship for only PFOS and birth weight (see Supplemental Material, Figure S2), which appeared to be an artifact of differing exposure ranges and a slight positive slope for Poland and highly negative slope for Greenland (*p* = 0.52 and *p* = 0.001, respectively, for linear terms). Only minor differences in coefficients were observed when wet weight rather than lipid-standardized organochlorines were modeled (Table 3, footnote; see also Supplemental Material, Table S4) and when unpenalized models were additionally adjusted for covariates with missing data (maternal education, season, and seafood) (data not shown).

## Discussion

In this study of mother–newborn pairs from Greenland, Poland, and Ukraine, prenatal exposure to a DEHP metabolite (MEHHP), PFOA, and *p,p´*-DDE were independently associated with lower birth weight in term newborns. This study considers one of the largest numbers of environmental contaminants to date, in relation to birth weight, and has one of the larger sample sizes.

To overcome the challenge of assessing associations for correlated exposures, we used a multi-pollutant penalized regression approach to select a subset of the most predictive environmental exposure variables. Elastic net regression forces coefficients of the least predictive variables to zero. We inputted the exposures selected using elastic net regression (MEHHP, MOiNP, PFOA, and *p,p´-*DDE) into multiple-exposure unpenalized regression models to obtain estimates of the linear associations between exposures and term birth weight which were not shrunken (or were unpenalized).

Selection based on single-exposure, unpenalized OLS models somewhat coincided with elastic net selection. However, in these models, each exposure was not mutually adjusted for other exposures, and some associations likely reflect false positives that are “tracking” with correlated exposure(s) truly associated with the outcome. For instance, the magnitude of associations for PFOS and PCB-153 decreased markedly when modeled with other exposures (data not shown). We decided to use a penalized regression approach because we expected, based on the correlation matrix, that an OLS regression model including all exposures would suffer from multicollinearity. *A posteriori*, we confirmed this. The variance inflation factor exceeded 3 for 6 of the 16 exposures, and the direction of the coefficient flipped from negative to positive for 4 exposures (data not shown). Using a more conventional backward–forward stepwise regression yielded generally similar selection results as the elastic net models (data not shown). However, selection accuracy would theoretically be expected to suffer given that exposures are not mutually adjusted at each step. Elastic net would be expected to have even more power in a data scenario with a higher number of exposures and might offer a complementary selection strategy to the single-exposure environment-wide association study (EWAS) approach.

There are caveats to applying a prediction method to an etiologic research question. With two highly correlated variables, elastic net will select a slightly more predictive variable, and shrink the coefficient of the other variable, which may be etiologically relevant. Nevertheless, with conventional (unpenalized) regression modeling, the strength of an association is usually also interpreted as having etiologic implications. Selection based on minimizing prediction error in CV, as performed in the present study, has been shown to overselect predictors. Thus we also applied the recently proposed covariance test for postselection inference (Lockhart et al. 2014), which has been shown to have lower power than CV-based selection but also a lower FDR (Sabourin et al. 2015). This resulted in consistent, although more conservative (sparser), selection than CV, and indicated strongest support for associations between MEHHP and *p,p´-*DDE and birth weight (Table 2). Other selection strategies have been proposed, such as assessing the stability of selection via subsampling of data (Meinshausen and Bühlmann 2010), which also indicated that MEHHP and *p,p´*-DDE were most robustly selected (data not shown). Improving the statistical inference of elastic net models, including explicitly addressing multiple testing, warrants further research and validation.

We restricted our analysis to term births because exposures may represent causally independent risk factors for preterm birth and growth restriction. Results did not differ appreciably upon inclusion of preterm births (data not shown). We did not have data on gestational weight gain, a proxy of lipid gain, which has been shown in simulations to confound associations between organochlorines and birth weight (Verner et al. 2013) but has also been suggested to partially mediate associations (Casas et al. 2015). Inclusion of gestational weight gain or estimated fat mass in a pooled analysis of a subset of the European cohorts with available data (*n* = 4,266) reduced the effect estimates for cord blood PCB-153 and birth weight by 48% and 34%, respectively (Casas et al. 2015). For PFASs, a lower rate of pregnancy-related plasma volume expansion or glomerular filtration increase might confound PFAS–birth weight associations (Morken et al. 2014).

Further, we did not have data on interpregnancy interval, gestational diabetes, preeclampsia, or detailed information on fish intake, possibly contributing to residual confounding. We adjusted for study population to limit bias due to unmeasured confounding, but a corollary is reduced contrast in exposure. We also cannot exclude the possibility that unmeasured (contaminant) exposures confound the observed associations. Furthermore, differential gestational timing of sampling across study populations may have led to less precise and biased coefficients.

The phthalate metabolites have shorter half-lives than the other contaminants. Braun et al. (2012) demonstrated high intraindividual variability of urinary ΣDEHP oxidative metabolites (intraclass correlation coefficient of 0.09) in a study with serial sampling of women throughout pregnancy. Thus, repeated measurements would have been preferable. Given that exposure assessment of the phthalates was relatively imprecise due to their short half-lives, leading to possibly attenuated estimates and a loss of statistical power, it is perhaps surprising that we identified a consistent association with MEHHP. Although most studies assessed exposure to phthalates using urine samples, several studies support that secondary oxidative metabolites can be reliably assessed in serum samples because they, unlike the primary monoesters, are not susceptible to lipase activity and external contamination by phthalate diesters in sampling devices and *ex vivo* conversion during analysis and storage (Frederiksen et al. 2010; Hart et al. 2014).

Most previous assessments of contaminant exposures and birth weight have used single-exposure, sometimes two-exposure, modeling. Two studies (*n* = 287 and 404) which tested 11 and 10 urinary phthalate (including DEHP) metabolites, respectively, found no associations for phthalates and some associations for phenols (Philippat et al. 2012; Wolff et al. 2008).

Of the larger studies (*n* ≥ 400–849) of environmental PFAS exposure and birth weight, one reported significant inverse associations for all PFASs evaluated (PFOS, PFOA, and PFHxS) (Maisonet et al. 2012); another reported nonsignificant inverse associations for PFOS and PFOA and birth weight *z*-scores (Whitworth et al. 2012); and two studies reported inverse associations for PFOS, but not for PFOA (Washino et al. 2009) or PFOA, PFNA, and PFUA (Chen et al. 2012). A large study within the Danish National Birth Cohort (*n* = 1,388), observed a mean PFOS plasma level of 35.3 ng/mL and PFOA level of 5.6 ng/mL, higher than levels in the present study; 12.9 and 1.7 ng/mL serum, respectively. A significant association was reported for PFOA (–10.63 g; 95% CI: –20.79, –0.47 per 1 ng/mL for all births, and –8.73 g; 95% CI: –19.53, 2.06 for term births) but not PFOS—in line with our findings—and the association for PFOA increased slightly in magnitude upon adjustment for PFOS (Fei et al. 2007). Comparisons of concentrations in paired maternal–cord blood samples, suggest that PFOA crosses the placental barrier more readily than PFOS, which may partly account for the observed dominance of PFOA estimates over PFOS (Fei et al. 2007). Inconsistent with this finding, in a community exposed to high levels of PFOA in Ohio, USA (*n* = 1,470), PFOA (geometric mean, 16.2 ng/mL) was not associated with term birth weight, whereas PFOS (geometric mean, 13.2 ng/mL) was inversely associated, albeit not consistently statistically significant across analyses, and coefficients remained stable in a two-pollutant model (Darrow et al. 2013).

Our findings for the organochlorines somewhat contradict those of a pooled analysis of European cohorts (*n* ~ 9,000), including the study populations of this study, although risk estimates displayed heterogeneity across cohorts. Cord serum PCB-153 (measured or estimated from maternal serum, whole blood, or breast milk levels) was significantly associated with reduced birth weight, but *p,p´-*DDE was not significantly associated: –194 g (95% CI: –314, –74) per 1 μg/L PCB-153 and –9 g (95% CI: –19, 7) per 1 μg/L *p,p´-*DDE. Associations were reported not to change in a two-pollutant model (Casas et al. 2015).

Postulated mechanisms include *a*) increased oxidative stress (Ferguson et al. 2014) and *b*) modulation of sex hormone or thyroid hormone homeostasis, which both play a role in development and growth velocities. Cord blood organochlorine compounds and maternal serum PFASs have been found to be inversely associated with thyroid hormone levels (Maervoet et al. 2007; Wang et al. 2014). There is also experimental evidence that PFASs interfere with lipid metabolism via activation of the peroxisome proliferator-activated receptor alpha (PPARα) (Wolf et al. 2012). Mid-pregnancy PFAS levels have been associated with high-density lipoprotein (HDL) and total cholesterol (Starling et al. 2014). In addition, a study of pregnant women found that bisphenol A and a few phthalates, including metabolites of DEHP, were associated with perturbations in biomarkers of angiogenesis linked to placental development and function, with potential adverse consequences for fetal growth (Ferguson et al. 2015).

Our results suggest that smoking during pregnancy and prepregnancy overweight/obesity may confer greater susceptibility to the effects of some contaminants on birth weight, although mechanisms underlying these potential interactions remain unclear. Casas et al. (2015) assessed effect measure modification for PCB-153 and birth weight, and reported stronger inverse associations for maternal prepregnancy overweight/obese, and smoking during pregnancy (although only the latter interaction was significant at *p* < 0.05); however, the evidence for potential effect modifiers is generally limited or inconsistent for contaminants assessed in the present analysis.

## Conclusions

We found indications that several environmental contaminant exposures, representing three chemical classes (phthalates, PFASs, and organochlorines), are independently associated with reduced birth weight, with possible implications for health trajectories. Cautious interpretation is warranted in light of possible confounding due to pregnancy-related pharmacokinetic issues and unmeasured contaminant exposures. We used penalized elastic net regression to assess a mixture of environmental contaminants; this modeling approach may prove useful for similar environmental epidemiology analyses of multiple (correlated) exposures.

## Supplemental Material

(601 KB) PDF
