# Supplementary material for: Prenatal Phthalate, Perfluoroalkyl Acid, and Organochlorine Exposures and Term Birth Weight in Three Birth Cohorts: Multi-Pollutant Models Based on Elastic Net Regression
Source: Environ Health Perspect. 2015 Jun 26;124(3):365–72. doi: 10.1289/ehp.1408933 (PMC4786980; doi:10.1289/ehp.1408933)
Supplement: (601 KB) PDF [file ehp.1408933.s001.acco.pdf]

Environ Health Perspect
DOI: 10.1289/ehp.1408933

Note to Readers: *EHP* strives to ensure that all journal content is accessible to all readers. However, some figures and Supplemental Material published in *EHP* articles may not conform to 508 standards due to the complexity of the information being presented. If you need assistance accessing journal content, please contact ehp508@niehs.nih.gov. Our staff will work with you to assess and meet your accessibility needs within 3 working days.

Supplemental Material

Prenatal Phthalate, Perfluoroalkyl Acid, and Organochlorine Exposures and Term Birth Weight in Three Birth Cohorts: Multi-Pollutant Models Based on Elastic Net Regression

Virissa Lenters, Lützen Portengen, Anna Rignell-Hydbom, Bo A.G. Jönsson, Christian H. Lindh, Aldert H. Piersma, Gunnar Toft, Jens Peter Bonde, Dick Heederik, Lars Rylander, and Roel Vermeulen

Table of Contents

Figure S1. Directed acyclic graph to describe the authors' view of the relationships between the contaminant exposures, covariates and birth weight.

Table S1. Serum concentrations of exposure biomarkers in pregnant women from Greenland (n=513), Warsaw, Poland (n=180), and Kharkiv, Ukraine (n=557), 2002–2004.

Table S2. Spearman correlation coefficients between exposure biomarkers[a] in pooled samples, and per study population.

Table S3. Adjusted associations (β [95% CI]) between term birth weight and selected exposures and demographic, reproductive, and lifestyle factors.

Table S4. Single-exposure unpenalized OLS-regression models for term birth weight (n=1250).

Table S5. Assessment of potential effect modification of the associations between contaminant exposures and term birth weight.

Figure S2. Generalized additive models for the single-pollutant exposure–outcome relationship for PFOS and term birth weight, fitted with a smoothing spline term for PFOS (with restricted maximum likelihood estimation).

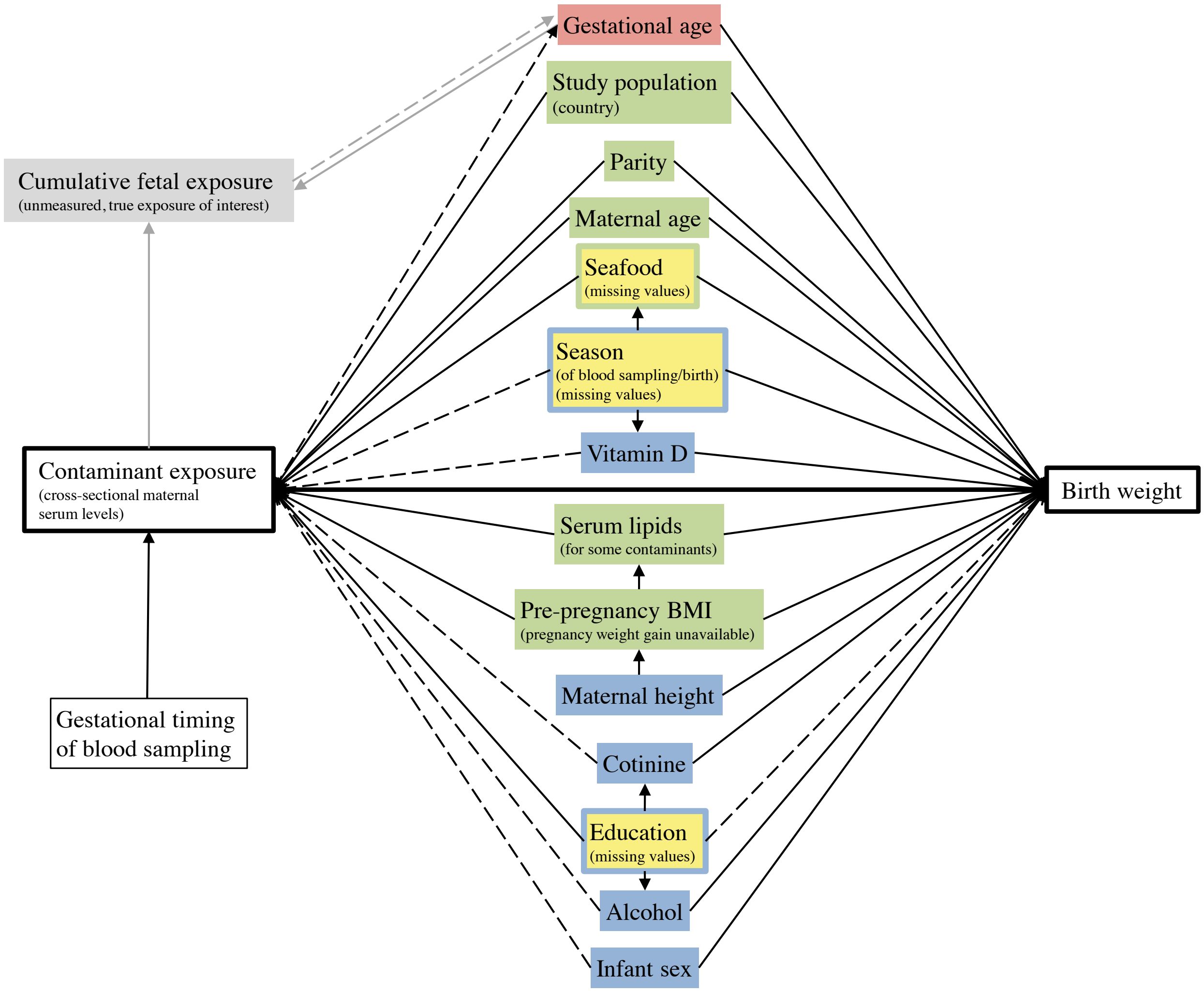

Figure S1. Directed acyclic graph to describe the authors' view of the relationships between the contaminant exposures, covariates and birth weight. The minimal sufficient adjustment set for estimating the total effect of contaminants on birth weight: study population, maternal age, pre-pregnancy body mass index (BMI) and parity (green boxes). For birth weight, serum lipids likely represent a confounder for the organochlorines (PCB-153 and *p,p*'-DDE), but not the PFASs or phthalate metabolites. Data on gestational weight gain—a proxy for gain in fat mass during pregnancy, which is an important confounder—was not available. Exposure of the fetus to environmental contaminants (grey box) was assessed by a proxy measure, cross-sectional maternal serum concentrations. While gestational age has an effect on cumulative fetal exposures, it is has no direct causal effect on cross-sectional maternal concentrations. However,

fetal/maternal concentrations may have a causal effect on gestational age. Thus gestational age was considered a potential intermediate, and was added separately to the models. Seafood consumption represents a potentially important confounder for some contaminants (e.g., PCB-153); however, this variable represents consumption of different types of seafood/fish in the different study populations (countries). Furthermore, seafood consumption, along with season and maternal education, had a large number of missing values, so these variables were only included in sensitivity analyses (yellow boxes). The relationships between some covariates and contaminants and birth weight are less certain (denoted by a dashed line), and these covariates (blue boxes) were added in a secondary analysis, ‘further adjusted models’; this adjustment set may represent overadjustment or unnecessary adjustment. This is a simplified representation of inter-relationships. Other permutations of the model could be argued. For example, maternal BMI affects measured contaminant levels, but may also represent an intermediate, causally affected by contaminant levels.

Table S1. Serum concentrations[a] of exposure biomarkers in pregnant women from Greenland (n=513), Warsaw, Poland (n=180), and Kharkiv, Ukraine (n=557), 2002–2004.

**Analyte** (ng/mL)	**CV** (%)[b]	**LOD** (ng/mL)[c]	**% >LOD** (n=1250)	**GR** 5 P	**GR** 50 P	**GR** 95 P	**PL** 5 P	**PL** 50 P	**PL** 95 P	**UA** 5 P	**UA** 50 P	**UA** 95 P	GR *vs.* PL *p*-value[d]	GR *vs.* UA *p*-value[d]	PL *vs.* UA *p*-value[d]
Phthalate metabolites															
MEHHP	15, 17	0.01	100.0	0.24	0.68	2.36	0.17	0.41	1.07	0.11	0.46	2.94	<0.001	<0.001	0.011
MEOHP	12, 19	0.02	99.3	0.05	0.12	0.28	0.04	0.10	0.23	0.04	0.11	0.38	<0.001	0.062	0.011
MECPP	15, 18	0.007	100.0	0.25	0.58	2.00	0.34	0.85	2.22	0.35	0.93	4.00	<0.001	<0.001	0.034
ΣDEHPom[e] (nmol/L)	–	–	–	2.37	4.83	13.64	2.06	4.56	9.67	1.99	5.53	21.17	0.091	0.003	<0.001
ΣDEHPom[e]	–	–	–	0.71	1.46	4.11	0.62	1.38	2.92	0.60	1.67	6.38	0.091	0.003	<0.001
MHiNP	14, 16	0.01	95.5	0.07	0.24	0.79	0.02	0.11	0.54	<LOD	0.04	0.48	<0.001	<0.001	<0.001
MOiNP	16, 20	0.005	90.2	<LOD	0.02	0.07	0.005	0.02	0.06	<LOD	0.01	0.24	0.047	0.078	0.003
MCiOP	13, 14	0.03	100.0	0.07	0.23	3.25	0.11	0.25	0.88	0.05	0.21	4.57	0.062	0.221	0.011
ΣDiNPom[e] (nmol/L)	–	–	–	0.68	1.65	12.18	0.67	1.37	3.88	0.28	0.84	18.48	<0.001	<0.001	<0.001
ΣDiNPom[e]	–	–	–	0.22	0.52	3.86	0.21	0.44	1.23	0.09	0.27	5.86	<0.001	<0.001	<0.001
Perfluoroalkyl acids															
PFHxS	14, 18	0.02	100.0	0.99	2.05	5.07	0.97	2.28	5.95	0.45	1.56	4.09	0.058	<0.001	<0.001
PFHpA	11, 15	0.02	82.4	<LOD	0.05	0.15	0.02	0.11	0.60	<LOD	0.03	0.13	<0.001	<0.001	<0.001
PFOS	9, 11	0.2	100.0	10.23	20.09	49.47	4.38	7.81	12.40	2.27	5.04	9.48	<0.001	<0.001	<0.001
PFOA	6, 10	0.04	100.0	0.78	1.84	3.55	1.34	2.51	4.36	0.45	0.96	2.10	<0.001	<0.001	<0.001
PFNA	13, 13	0.03	100.0	0.33	0.69	1.99	0.30	0.56	1.34	0.30	0.61	1.37	<0.001	<0.001	0.033
PFDA	12, 13	0.03	99.9	0.16	0.40	1.18	0.10	0.22	0.45	0.07	0.16	0.34	<0.001	<0.001	<0.001
PFUnDA	19, 21	0.04	98.3	0.17	0.70	2.54	0.06	0.13	0.25	0.06	0.16	0.50	<0.001	<0.001	<0.001
PFDoDA	11, 15	0.04	72.4	0.04	0.13	0.40	<LOD	0.05	0.11	<LOD	0.04	0.11	<0.001	<0.001	0.194
Organochlorines															
PCB-153	10, 18	0.05	95.0	0.15	0.77	3.62	<LOD	0.10	0.29	0.06	0.19	0.53	<0.001	<0.001	<0.001
PCB-153 (ng/g)	–	–	95.0	21.03	106.82	526.47	2.59	10.75	27.02	8.50	26.98	68.06	<0.001	<0.001	<0.001
p,p′-DDE	7, 11	0.1	99.3	0.39	2.21	9.53	1.06	3.29	9.48	2.02	4.56	11.27	<0.001	<0.001	<0.001
p,p′-DDE (ng/g)	–	–	99.3	47.78	302.22	1289.32	137.29	347.82	876.12	276.01	653.44	1657.00	0.017	<0.001	<0.001
Total lipids (g/L)	–	–	100.0	4.10	7.37	13.53	7.51	9.63	11.98	4.44	7.18	10.91	<0.001	0.174	<0.001

CV, coefficient of variation; DEHP, diethylhexyl phthalate; DiNP, diisononyl phthalate; GM, geometric mean; GR, Greenland; LOD, limit of detection; MCiOP, mono-(4-methyl-7-carboxyheptyl)phthalate; MECPP, mono-(2-ethyl-5-carboxypentyl); MEHHP, mono-(2-ethyl-5-hydroxylhexyl) phthalate; MEOHP, mono-(2-ethyl-5-oxohexyl) phthalate; MHiNP, mono-(4-methyl-7-hydroxyloctyl)phthalate; MOiNP, mono-(4-methyl-7-oxo octyl)phthalate; P, percentile; PCB-153, 2,2′,4,4′,5,5′-hexachlorobiphenyl; PFDA, perfluorodecanoic acid; PFHxS, perfluorohexane sulfonic acid; PFHpA,

perfluoroheptanoic acid; PFOS, perfluorooctane sulfonic acid; PFOA, perfluorooctanoic acid; PFNA, perfluorononanoic acid; PFUnDA, perfluoroundecanoic acid; PFDoDA, perfluorododecanoic acid; PL, Poland; *p,p'*-DDE, 1,1-dichloro-2,2-bis(p-chlorophenyl)-ethylene; UA, Ukraine.

[a] Values below the LOD were imputed (see main text for details).

[b] CV for the between-day precision in 2 different quality control samples (n=76).

[c] LOD determined as the concentration corresponding to 3 times the standard deviation of the chemical blank signal.

[d] Pairwise comparisons of concentrations between study populations: Mann-Whitney U-test.

[e] Sum of DEHP or DiNP secondary oxidative metabolites, calculated as (1) the molar sum (nmol/L) and (2) corrected for molecular weight, based on the weighted average molecular weight (ng/mL).

Table S2. Spearman correlation coefficients between exposure biomarkers[a] in pooled samples, and per study population.

		MEHHP	MEOHP	MECPP	MHiNP	MOiNP	MCiOP	PFHxS	PFHpA	PFOS	PFOA	PFNA	PFDA	PFUnDA	PFDoDA	PCB-153	*p,p'*-DDE
**MEHHP**	**All**	1.00	0.75*	0.19*	0.28*	0.14*	0.15*	0.03*	0.03*	0.21*	0.07*	0.09*	0.17*	0.17*	0.11*	0.23*	-0.09*
	**GR**	1.00	0.65*	0.24*	0.32*	0.10*	0.05	-0.09*	0.02	-0.09*	0.01	-0.02	-0.06	-0.11*	-0.13*	-0.15*	-0.19*
	**PL**	1.00	0.62*	0.30*	0.24*	0.13	0.07	0.00	0.12	0.03	-0.01	0.07	0.00	-0.05	-0.04	0.11	0.01
	**UA**	1.00	0.85*	0.38*	0.11*	0.18*	0.26*	0.08	0.01	0.10*	0.09*	0.08	0.11*	-0.01	-0.10*	0.13*	0.10*
**MEOHP**	**All**		1.00	0.46*	0.17*	0.27*	0.24*	0.03*	0.03*	0.08*	0.04*	0.06*	0.08*	0.05*	0.02	0.12*	-0.01
	**GR**		1.00	0.53*	0.27*	0.26*	0.16*	-0.01	0.03	0.03	0.10*	0.02	0.03	-0.04	-0.02	-0.05	-0.08
	**PL**		1.00	0.50*	-0.06	0.25*	0.30*	0.15*	0.06	0.01	0.11	0.15*	0.11	0.00	-0.06	0.03	-0.03
	**UA**		1.00	0.54*	0.18*	0.31*	0.31*	0.03	0.04	0.07	0.04	0.04	0.06	-0.03	-0.09*	0.14*	0.11*
**MECPP**	**All**			1.00	-0.08*	0.34*	0.34*	-0.05*	-0.02*	-0.28*	-0.12*	-0.05*	-0.21*	-0.24*	-0.22*	-0.21*	0.15*
	**GR**			1.00	0.12*	0.33*	0.30*	0.05	0.06	0.04	0.07	-0.02	0.02	-0.02	0.00	0.01	-0.02
	**PL**			1.00	-0.03	0.34*	0.47*	0.08	0.07	0.02	0.07	0.12	0.05	0.12	0.04	-0.11	-0.09
	**UA**			1.00	0.31*	0.44*	0.4*1	-0.01	0.00	0.04	0.03	-0.03	0.00	-0.03	0.00	0.09*	0.08
**MHiNP**	**All**				1.00	0.36*	0.31*	0.12*	0.19*	0.56*	0.32*	0.07*	0.44*	0.45*	0.45*	0.34*	-0.34*
	**GR**				1.00	0.16*	0.17*	-0.11*	0.07	0.03	-0.02	0.03	0.05	0.01	0.05	-0.15*	-0.16*
	**PL**				1.00	0.16*	0.02	0.03	-0.06	0.10	-0.21*	-0.18*	-0.05	0.05	0.18*	0.09	0.15*
	**UA**				1.00	0.63*	0.53*	-0.08	0.04	0.02	0.02	0.03	0.04	0.09*	0.08*	0.03	-0.08
**MOiNP**	**All**					1.00	0.58*	-0.01	0.06*	0.01	0.08*	-0.04*	0.02	-0.01	0.03*	-0.04*	-0.08*
	**GR**					1.00	0.41*	-0.04	0.00	-0.12*	0.05	-0.14*	-0.11*	-0.15*	-0.04	-0.08	-0.08
	**PL**					1.00	0.58*	-0.03	0.04	0.02	0.06	0.06	0.06	0.15*	0.07	-0.03	-0.01
	**UA**					1.00	0.68*	-0.05	0.04	-0.02	0.04	0.01	0.05	0.01	0.07	-0.02	-0.05
**MCiOP**	**All**						1.00	0.11*	0.10*	0.04*	0.13*	0.05*	0.07*	0.02	0.02	0.00	-0.01
	**GR**						1.00*	0.09	0.08	0.07*	0.09	0.01	0.06	0.02	0.06	0.03	0.02
	**PL**						1.00	0.26*	0.17*	0.03	0.13	0.12	0.13	0.08	-0.01	-0.18*	-0.05
	**UA**						1.00	0.08	0.05	0.03	0.16*	0.09*	0.08*	0.02	-0.03	0.03	0.04
**PFHxS**	**All**							1.00	0.17*	0.34*	0.34*	0.22*	0.35*	0.23*	0.24*	0.15*	-0.01
	**GR**							1.00	0.03	0.37*	0.19*	0.31*	0.41*	0.30*	0.34*	0.28*	0.27*
	**PL**							1.00	0.10	0.27*	0.17*	0.18*	0.19*	0.07	0.06	0.09	0.09
	**UA**							1.00	0.10*	0.24*	0.26*	0.17*	0.19*	0.14*	0.06	0.03	0.04
**PFHpA**	**All**								1.00	0.26*	0.44*	0.22*	0.25*	0.11*	0.09*	0.02*	-0.06*
	**GR**								1.00	0.21*	0.20*	0.27*	0.19*	0.17*	0.10*	0.20*	0.15*
	**PL**								1.00	0.22*	0.44*	0.53*	0.38*	0.25*	0.07	0.14	0.08
	**UA**								1.00	0.17*	0.33*	0.16*	0.11*	0.06	-0.05	0.01	0.09*

		MEHHP	MEOHP	MECPP	MHiNP	MOiNP	MCiOP	PFHxS	PFHpA	PFOS	PFOA	PFNA	PFDA	PFUnDA	PFDoDA	PCB-153	*p,p'*-DDE
**PFOS**	**All**									1.00	0.61*	0.42*	0.78*	0.74*	0.64*	0.62*	-0.24*
	**GR**									1.00	0.50*	0.74*	0.72*	0.64*	0.48*	0.52*	0.50*
	**PL**									1.00	0.55*	0.58*	0.47*	0.37*	0.08	0.20*	0.24*
	**UA**									1.00	0.53*	0.54*	0.52*	0.41*	0.11*	0.23*	0.14*
**PFOA**	**All**										1.00	0.30*	0.50*	0.29*	0.26*	0.15*	-0.19*
	**GR**										1.00	0.24*	0.15*	-0.03	-0.02	0.08	0.12*
	**PL**										1.00	0.70*	0.55*	0.24*	0.06	0.19*	0.17*
	**UA**										1.00	0.46*	0.47*	0.30*	0.06	0.14*	0.14*
**PFNA**	**All**											1.00	0.60*	0.48*	0.31*	0.36*	0.27*
	**GR**											1.00	0.85*	0.83*	0.63*	0.65*	0.61*
	**PL**											1.00	0.79*	0.47*	0.16*	0.15*	0.10
	**UA**											1.00	0.53*	0.44*	0.08	0.20*	0.14*
**PFDA**	**All**												1.00	0.77*	0.67*	0.56*	-0.08*
	**GR**												1.00	0.86*	0.74*	0.62*	0.60*
	**PL**												1.00	0.54*	0.30*	0.11	0.12
	**UA**												1.00	0.57*	0.26*	0.16*	0.09*
**PFUnDA**	**All**													1.00	0.71*	0.67*	-0.09*
	**GR**													1.00	0.75*	0.65*	0.60*
	**PL**													1.00	0.48*	0.18*	0.11
	**UA**													1.00	0.39*	0.15*	0.06
**PFDoDA**	**All**														1.00	0.56*	-0.13*
	**GR**														1.00	0.50*	0.46*
	**PL**														1.00	-0.05	0.01
	**UA**														1.00	0.08*	-0.01
**PCB-153**	**All**															1.00	0.19*
	**GR**															1.00	0.92*
	**PL**															1.00	0.54*
	**UA**															1.00	0.50*
***p,p'*-DDE**	**All**																1.00
	**GR**																1.00
	**PL**																1.00
	**UA**																1.00

All represents pooled study populations; GR, Greenland; PL, Poland; UA, Ukraine.
[a] Phthalates and PFASs in ng/mL serum, and organochlorines in ng/g lipids.
* $p<0.05$; considered statistically significant.

Table S3. Adjusted associations (*β* [95% CI]) between term birth weight and selected exposures and demographic, reproductive, and lifestyle factors.

**Covariate (increment)**	**Term birth weight** (g)	**ΣDEHPom** (nmol/L)	**ΣDiNPom** (nmol/L)	**PFOS** (ng/mL)	**PFOA** (ng/mL)	**PCB-153** (ng/g)	***p,p*´-DDE** (ng/g)
***Model 1** (n=1250)*							
Intercept	3707.7 (3620.8, 3794.7)*	1.72 (1.59, 1.85)*	0.64 (0.43, 0.86)*	3.05 (2.96, 3.14)*	0.73 (0.63, 0.83)*	4.47 (4.30, 4.64)*	5.48 (5.31, 5.64)*
Study population (ref.: Greenland)							
Poland	-97.0 (-187.2, 6.9)*	-0.22 (-0.36, 0.08)*	-0.29 (-0.51, 0.06)*	-1.03 (-1.12, 0.93)*	0.19 (0.09, 0.29)*	-2.41 (-2.58, 2.24)*	0.43 (0.26, 0.60)*
Ukraine	-235.8 (-301.1, 170.6)*	0.06 (-0.04, 0.16)	-0.45 (-0.61, 0.29)*	-1.45 (-1.52, 1.38)*	-0.74 (-0.81, 0.67)*	-1.35 (-1.47, 1.22)*	0.96 (0.84, 1.08)*
Maternal total lipids (5.11 g/L)	33.0 (-14.9, 80.9)	0.04 (-0.03, 0.11)	0.11 (-0.01, 0.23)	-0.06 (-0.12, 0.01)*	-0.12 (-0.17, 0.07)*	-0.27 (-0.37, 0.18)*	-0.40 (-0.49, 0.31)*
Gestational age (2.45 weeks)	336.2 (289.2, 383.2)*	-0.03 (-0.11, 0.04)	-0.02 (-0.14, 0.09)	-0.06 (-0.11, 0.01)*	-0.02 (-0.07, 0.03)	-0.05 (-0.14, 0.04)	-0.12 (-0.21, 0.03)*
Infant sex (ref.: male): female	-116.2 (-161.5, 70.9)*	-0.02 (-0.09, 0.05)	-0.04 (-0.15, 0.07)	-0.03 (-0.07, 0.02)	-0.01 (-0.06, 0.04)	0.02 (-0.06, 0.11)	-0.01 (-0.10, 0.08)
Maternal age (years)[a] (ref: 18–26)							
27–31	53.2 (-5.7, 112.1)	0.09 (0.00, 0.18)*	0.13 (-0.02, 0.27)	0.07 (0.00, 0.13)*	0.05 (-0.02, 0.12)	0.24 (0.13, 0.36)*	0.17 (0.06, 0.28)*
32–45	14.5 (-51.4, 80.3)	0.09 (-0.01, 0.19)	0.03 (-0.14, 0.19)	0.15 (0.08, 0.22)*	0.11 (0.04, 0.19)*	0.53 (0.40, 0.65)*	0.34 (0.21, 0.46)*
Pre-pregnancy BMI (8.62 kg/m^2)	192.8 (143.9, 241.6)*	-0.09 (-0.16, 0.01)*	-0.11 (-0.23, 0.01)	0.05 (-0.00, 0.10)	0.04 (-0.01, 0.10)	-0.12 (-0.22, 0.03)*	0.02 (-0.07, 0.11)
Maternal height (12.93 cm)	141.5 (94.0, 188.9)*	-0.04 (-0.12, 0.03)	0.04 (-0.08, 0.15)	-0.00 (-0.05, 0.05)	0.06 (0.00, 0.11)*	-0.16 (-0.25, 0.07)*	-0.14 (-0.23, 0.05)*
Parity (ref.: nulliparous): multiparous	102.6 (46.8, 158.3)*	-0.05 (-0.14, 0.04)	0.02 (-0.12, 0.15)	-0.08 (-0.13, 0.02)*	-0.23 (-0.29, 0.17)*	-0.11 (-0.21, 0.00)	-0.07 (-0.18, 0.03)
Serum cotinine (113.51 ng/mL)	-147.6 (-199.0, -96.1)*	-0.03 (-0.11, 0.05)	0.29 (0.16, 0.42)*	0.04 (-0.01, 0.10)	-0.02 (-0.08, 0.04)	0.21 (0.11, 0.31)*	0.22 (0.12, 0.31)*
Vitamin D (22.05 ng/mL)	13.2 (-35.3, 61.6)	0.00 (-0.07, 0.08)	0.01 (-0.11, 0.12)	0.13 (0.08, 0.19)*	0.05 (-0.00, 0.10)	0.22 (0.13, 0.31)*	0.12 (0.03, 0.21)*
Alcohol intake (ref.: <7): ≥7 drinks/weeks[b]	34.5 (-61.9, 130.8)	-0.17 (-0.32, 0.02)*	0.04 (-0.20, 0.27)	0.11 (0.01, 0.22)*	0.00 (-0.11, 0.11)	0.25 (0.07, 0.44)*	0.28 (0.10, 0.47)*
Adjusted R^2	*0.32*	*0.02*	*0.07*	*0.71*	*0.41*	*0.58*	*0.29*
***Model 2:** Model 1 + the following covariates (n=1122)*							
Maternal education (ref.: none): some post-secondary	-14.2 (-68.0, 39.6)	0.09 (0.01, 0.17)*	-0.05 (-0.18, 0.08)	0.02 (-0.03, 0.08)	0.09 (0.04, 0.15)*	-0.08 (-0.19, 0.02)	-0.01 (-0.11, 0.09)
Season of blood sampling (ref.: Oct.–March): April–Sept.	6.0 (-45.6, 57.7)	-0.18 (-0.26, 0.10)*	-0.25 (-0.37, 0.13)*	0.07 (0.02, 0.12)*	-0.01 (-0.07, 0.04)	0.09 (-0.01, 0.18)	0.09 (-0.00, 0.19)
Fish/seafood (3.03 days/week)[b]	-20.5 (-37.1, 3.9)*	-0.01 (-0.03, 0.02)	0.05 (0.01, 0.09)*	0.05 (0.03, 0.07)*	0.02 (0.01, 0.04)*	0.07 (0.04, 0.10)*	0.06 (0.03, 0.09)*
Adjusted R^2	*0.32*	*0.04*	*0.09*	*0.72*	*0.44*	*0.60*	*0.30*

ref, Reference category.

Regression coefficients (*β*) are estimated from multivariable OLS linear regression models of term birth weight or ln-exposures on (Model 1) covariates with no missing data, and (Model 2) with the addition of covariates with missing data. Continuous covariates were mean-centered and rescaled to 2 times their standard deviations (increment). Variance inflation factors (VIF, a measure of multicollinearity) were all <1.50, except for study population (VIF=2.48) in term birth weight models.

* Considered statistically significant: The 95% confidence interval for *β* did not include unity.

[a] These cutoffs for age yield a low AIC for a model of birth weight and age, which most closely matches the AIC from a GAM model with age fitted with a smoothing spline (best fit was an inverted U-shape).

[b] With reference to period attempting to conceive.

Table S4. Single-exposure unpenalized OLS-regression models for term birth weight (n=1250).

Exposure	Geometric Mean	Ln, 2-SD[a]	Adjusted β_{OLS} (95% CI)	*p*-value	Plus gestational age β_{OLS} (95% CI)	*p*-value	Further adjusted β_{OLS} (95% CI)	*p*-value
MEHHP (ng/mL)	0.559	1.700	-73.89 (-125.96, -21.81)	0.006*	-72.66 (-120.92, -24.39)	0.003*	-63.63 (-110.44, -16.83)	0.008*
MEOHP (ng/mL)	0.113	1.293	-58.72 (-109.66, -7.79)	0.024*	-48.39 (-95.65, -1.12)	0.045	-44.75 (-90.45, 0.96)	0.055
MECPP (ng/mL)	0.813	1.421	-12.15 (-65.57, 41.26)	0.656	-2.82 (-52.36, 46.73)	0.911	-12.52 (-60.44, 35.39)	0.609
ΣDEHPom (mol/mL)	5.430	1.265	-60.48 (-111.50, -9.46)	0.020*	-52.50 (-99.83, -5.17)	0.030	-53.23 (-98.98, -7.47)	0.023
MHiNP (ng/mL)	0.096	2.735	28.60 (-34.90, 92.10)	0.378	28.78 (-30.09, 87.65)	0.338	33.55 (-23.48, 90.58)	0.249
MOiNP (ng/mL)	0.016	2.219	34.93 (-15.60, 85.47)	0.176	34.04 (-12.82, 80.89)	0.155	26.11 (-19.17, 71.39)	0.259
MCiOP (ng/mL)	0.275	2.321	-10.52 (-61.09, 40.06)	0.684	-3.85 (-50.75, 43.05)	0.872	11.79 (-33.97, 57.54)	0.614
ΣDINPom (mol/mL)	1.471	2.074	-0.69 (-52.90, 51.52)	0.979	6.70 (-41.72, 55.12)	0.786	20.75 (-26.28, 67.78)	0.387
PFHxS (ng/mL)	1.842	1.241	-47.74 (-101.07, 5.59)	0.080	-11.39 (-61.15, 38.37)	0.654	-6.28 (-55.18, 42.61)	0.801
PFHpA (ng/mL)	0.047	1.837	-10.17 (-66.19, 45.84)	0.722	-11.12 (-63.05, 40.82)	0.675	-7.05 (-57.18, 43.08)	0.783
PFOS (ng/mL)	9.357	1.600	-114.36 (-206.81, -21.91)	0.015*	-69.53 (-155.59, 16.53)	0.114	-68.84 (-152.90, 15.22)	0.109
PFOA (ng/mL)	1.421	1.175	-68.94 (-134.25, -3.63)	0.039	-61.06 (-121.63, -0.49)	0.048	-78.52 (-137.01, -20.03)	0.009*
PFNA (ng/mL)	0.652	1.028	-78.62 (-130.57, -26.66)	0.003*	-60.92 (-109.20, -12.63)	0.014*	-44.67 (-92.04, 2.69)	0.065
PFDA (ng/mL)	0.245	1.397	-103.28 (-169.84, -36.72)	0.002*	-64.80 (-126.87, -2.73)	0.041	-43.93 (-104.83, 16.97)	0.158
PFUnDA (ng/mL)	0.275	2.099	-102.04 (-174.80, -29.28)	0.006*	-65.99 (-133.74, 1.77)	0.057	-37.15 (-103.86, 29.56)	0.275
PFDoDA (ng/mL)	0.068	1.672	-83.97 (-150.78, -17.16)	0.014*	-35.64 (-98.07, 26.79)	0.263	-24.40 (-86.33, 37.54)	0.440
PCB-153 (ng/g)	39.620	2.432	-143.74 (-218.92, -68.57)	0.000*	-121.09 (-190.93, -51.24)	0.001*	-78.01 (-147.15, -8.88)	0.027
p,p′-DDE (ng/g)	419.856	1.823	-137.24 (-193.92, -80.56)	0.000*	-103.11 (-156.04, -50.17)	0.000*	-73.02 (-125.21, -20.83)	0.006*
PCB-153 (ng/mL)[b]	0.299	2.331	-141.82 (-216.21, -67.43)	0.000*	-103.75 (-171.60, -35.89)	0.003*	-65.25 (-132.29, 1.80)	0.057
p,p′-DDE (ng/mL)[b]	3.144	1.774	-134.91 (-191.88, -77.95)	0.000*	-93.14 (-145.99, -40.29)	0.001*	-64.83 (-116.78, -12.89)	0.015

OLS, ordinary least squares

Regression coefficients (β_{OLS}) represent the change in birth weight (g) for term infants per 2-standard deviation (SD) increase in natural-log (ln)–transformed exposure concentration. To convert the β_{EN} or β_{OLS} presented per 2-SD increase in ln-transformed exposure to a β coefficient per 1 unit increase in ln-exposure, apply the formula: (1 / 2-SD) * β. Adjusted models were adjusted for study population (Poland, Ukraine vs. Greenland); maternal age (27-31 and 32-45 vs. 18-26); pre-pregnancy BMI (kg/m^2); and parity (multiparous vs. nulliparious). Further adjusted models were additionally adjusted for gestational age (weeks); infant sex (female vs. male); maternal height (cm); alcohol (≥ drinks/week); cotinine (ng/mL); and vitamin D (ng/mL).

[a] 2 times the SD of ln–transformed concentrations (used for scaling in analyses).

[b] Wet weight PCB-153 and *p,p*′-DDE models were additionally adjusted for total lipids (g/L).

* *p*-value (two-sided) considered statistically significant at a false discovery rate (FDR)<5% (*q*-value <0.05) for the 16 exposures tested in the primary analysis; and significant at FDR<5% for all 20 exposures tested for the additional analysis, including summed phthalate metabolites [ΣDEHPom (mol/mL) and ΣDiNPom (mol/mL)] and wet weight organochlorines [PCB-153 (ng/mL) and *p,p*′-DDE (ng/mL)].

Table S5. Assessment of potential effect modification of the associations between contaminant exposures and term birth weight.

Potential modifier	n	MEHHP (1.70 ng/mL) β_{OLS} (95% CI)	MOiNP (2.22 ng/mL) β_{OLS} (95% CI)	PFOA (1.18 ng/mL) β_{OLS} (95% CI)	*p,p´*-DDE (1.82 ng/g) β_{OLS} (95% CI)
**Base model**	1250	-86.75 (-139.18, -34.32)*	45.85 (-4.84, 96.54)	-42.77 (-108.19, 22.65)	-134.73 (-191.93, -77.53)*
**Study population**					
Greenland	513	-118.80 (-234.15, -3.45)*	-10.76 (-121.04, 99.51)	-94.50 (-225.36, 36.37)	-153.00 (-234.70, -71.30)*
Poland	180	-134.24 (-315.62, 47.14)	148.65 (-10.48, 307.78)	60.21 (-118.31, 238.73)	-290.86 (-472.79, -108.92)*
Ukraine	557	-79.13 (-136.09, -22.16)*	57.07 (1.72, 112.42)*	-45.56 (-123.43, 32.32)	-42.42 (-148.4, 63.55)
p-interaction[a]		0.728	0.211	0.463	0.104
**Infant sex**					
Female	593	-97.53 (-170.53, -24.53)*	58.12 (-13.17, 129.40)	-32.76 (-123.77, 58.26)	-171.24 (-251.97, -90.52)*
Male	657	-70.73 (-145.35, 3.88)	42.10 (-29.10, 113.31)	-38.05 (-131.45, 55.35)	-111.20 (-191.02, -31.38)*
p-interaction[a]		0.946	0.910	0.835	0.263
**Pre-pregnancy BMI**					
<25 kg/m^2	972	-102.97 (-160.13, -45.81)*	61.39 (6.23, 116.55)*	-33.39 (-105.51, 38.73)	-82.32 (-150.81, -13.84)*
≥25 kg/m^2	278	-45.07 (-176.79, 86.66)	-53.12 (-181.46, 75.22)	-99.23 (-255.31, 56.85)	-246.81 (-352.90, -140.72)*
p-interaction[a]		0.169	0.158	0.759	0.029**
**Smoking: serum cotinine**					
Non-smoker: <5 ng/mL	851	-68.83 (-127.20, -10.46)*	34.91 (-19.51, 89.33)	-36.93 (-109.75, 35.88)	-47.58 (-118.22, 23.06)
Smoker: ≥5 ng/mL	399	-155.43 (-261.12, -49.74)*	64.90 (-48.19, 177.98)	-80.35 (-211.94, 51.23)	-186.32 (-284.07, -88.57)*
p-interaction[a]		0.025**	0.446	0.033**	0.198

The primary-selected exposures from elastic net modeling (see main text) were modeled in multiple-exposure unpenalized OLS regression models. Regression coefficients (β_{OLS}) represent the change in birth weight (g) for term infants per 2-SD increase in ln-transformed exposure concentration for the pooled study populations (increment indicated in the table heading). Models were adjusted for the minimal adjustment set (maternal age, pre-pregnancy BMI, parity, and study population), plus the potential modifier. Variance inflation factors (VIF) were <2.00 for all terms, across stratified models, except for study population (VIF=2.21─3.74 across models).

[a] *p*-values for interaction were calculated from a likelihood ratio test comparing multiple-exposure OLS models with and without the cross-product interaction term (exposure x potential modifier).

* Considered significant: The 95% confidence interval (CI) for β_{OLS} did not include unity.

** *p*-interaction < 0.05.

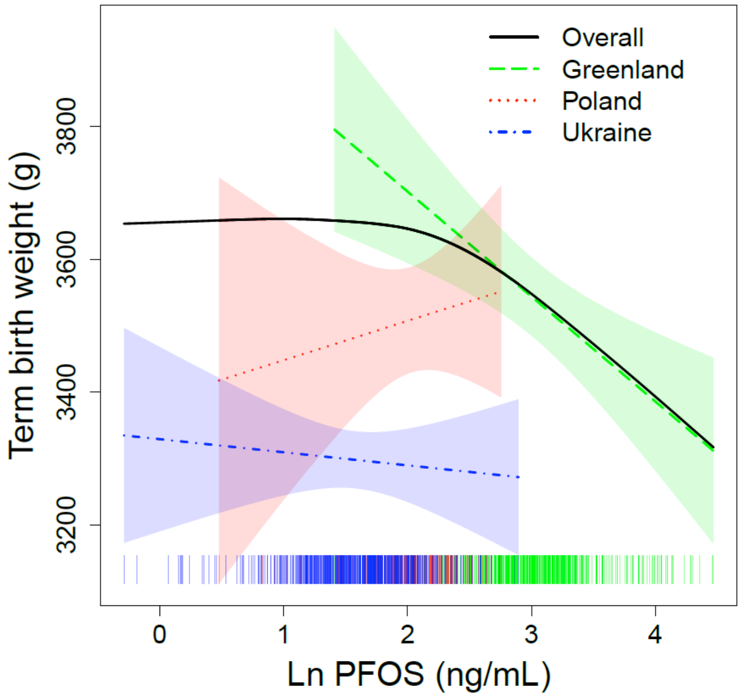

Figure S2. Generalized additive models for the single-pollutant exposure–outcome relationship for PFOS and term birth weight, fitted with a smoothing spline term for PFOS (with restricted maximum likelihood estimation). The other exposure and term birth weight models did not show evidence of significant non-linearity. The models were adjusted for study population (overall model), maternal age (categorical), pre-pregnancy BMI, and parity (as in the primary analyses). Predicted functions, with BMI set at the mean (22.89 kg/m2) and fixed at 18–26 years of age, and nulliparous, are presented: three population-specific exposure–outcome relationships (dashed lines) and 95% confidence intervals (shaded), and an overall exposure–outcome relationship for the pooled analysis, plotted at the Greenland-specific intercept (solid black line). Rug plots display the density of the PFOS exposure biomarker data.
